# The Major Domains of Comprehensive Assessment Tools for Older Adults Requiring Home-Based Aged Care Services: A Systematic Review

**DOI:** 10.3390/healthcare12232468

**Published:** 2024-12-06

**Authors:** Weiwei Fang, Hai Phung, Richard Olley, Patricia Lee

**Affiliations:** 1 School of Medicine and Dentistry, Griffith University, Gold Coast, QLD 4215, Australia; weiwei.fang@griffithuni.edu.au (W.F.); hai.n.phung@griffith.edu.au (H.P.); r.olley@griffith.edu.au (R.O.); 2 Australian College of Health Service Management, Gladesville, NSW 2111, Australia; 3 Department of Medical Research, China Medical University Hospital, China Medical University, Taichung 404328, Taiwan

**Keywords:** older adults, geriatric assessment, home care services

## Abstract

Background/Objectives: The global population is aging rapidly, increasing the need for appropriate health care. Older people often prefer to remain in their homes for as long as possible as they age. Therefore, it is crucial to assess their overall health and understand the individualized care needs for developing tailored home care services. This systematic review aims to examine the major domains of a range of assessment tools used for older people receiving home care services. Methods: A systematic search of Medline and PsycINFO via Ovid, CINAHL via EBSCO, Web of Science, and Scopus was conducted to identify studies investigating assessment of older people requiring home care services. The literature findings were systematically synthesized and classified using the International Classification of Functioning, Disability and Health (ICF) by the World Health Organization (WHO). Results: A total of 32 studies were included in the systematic review. Three primary categories were identified based on the WHO ICF classification system: (1) body functions, (2) activities and participation, and (3) environmental factors. Body functions included physical functions and mental functions. Mobility, self-care, and domestic life were three major aspects within the ICF category of activities and participation. Regarding the environmental factors, support, relationships, and services provided to older people were commonly considered in the included studies. Among them, the most assessed domains were physical, psychological, cognitive, functional, and nutritional assessment. Conclusions: The synthesis of findings in this review reveals major domains in various assessment tools, contributing to the development of a comprehensive framework to guide the assessment for older people requiring home care services.

## 1. Introduction

The global population is aging rapidly, with nearly every nation experiencing an increase in both the number and proportion of older people among its population [1,2]. As of 2021, the population aged 65 and above stood at 761.3 million, with projections indicating that this figure will surpass 1.6 billion by 2050 [3]. Moreover, the pace of population aging is much faster than it was in the past. The aging of the global population is an undeniable demographic reality with profound implications for healthcare systems, social services, and policy planning.

As the older population continues to age and expand, they are facing challenges of living with multiple chronic conditions and disabilities, which is also known as multimorbidity [4,5,6]. For instance, in Germany, approximately 24% of people aged 70 to 85 years are estimated to have five or more diseases at the same time [6]. The presence of multimorbidity markedly impairs both functional abilities and cognitive functions [2,7,8]. Accordingly, older people often have more healthcare needs [9], which in turn places greater strain on healthcare systems and increases social and economic burdens [10,11].

There has been a growing trend toward aging in place, driven by older adults’ desire to maintain their independence and live in familiar, comfortable surroundings for as long as possible [12]. Government policies have actively encouraged aging in place. This is because home-based care is generally considered more cost-effective than institutional care [12,13]. In many countries, there is also an increasing concern about the shortage of skilled healthcare workers in aged care [12]. Additionally, older adults prefer to age at home, as it enables them to maintain their independence [13,14] and stay connected to their communities [15,16], as long as their health and abilities allow it. This trend has led to an increased demand for appropriate aged care services in home care settings [17,18].

Informal home care, i.e., unpaid care provided by family members motivated by familial affection, is the primary source of caring for seniors [13]. However, changes in traditional family dynamics over time have made informal care alone insufficient [6]. As a result, more older adults will need formal care services provided by professional aged care providers [19]. This is partly because the long-term viability of unpaid care is challenged by the substantial effects that extended caregiving responsibilities can have on caregivers’ physical and mental health, as well as their financial well-being [20]. It is also worth noting that certain types of home care services are beyond the scope of what families can provide [6]. Collectively, there is a challenge to meet the care and support needs of an aging population both now and in the future.

To support aging in place, it is crucial to ensure that older adults’ health and functional needs are met in the home environment, and that these needs are continually monitored and addressed. This makes standardized assessments essential. A comprehensive assessment of an individual’s health status, physical function, cognitive function, social support, and environmental factors is necessary to determine whether their current living situation is conducive to aging in place [21,22]. Additionally, regular assessments help identify emerging challenges or health changes that may affect the ability to live independently [23]. By adopting a structured, multidimensional assessment approach, care providers and policymakers can make informed decisions about the services and interventions necessary to support aging in place.

Care services tailored to the needs and preferences of older adults have emerged as an urgent public health priority. It is imperative that formal care and services are appropriate and accessible to enable seniors to age in their own homes. Older people often have multifactorial care needs [24]. To customize aged care services for older adults effectively, it is essential to firstly have an accurate and thorough understanding regarding their unique and evolving health status and functional abilities [13,17,24]. Enhanced understanding of these factors is fundamental for detecting overall health and changes in health [13], and for making necessary adjustments to the services provided [18], ensuring personalized and tailored care plans for optimal outcomes [10,17].

Comprehensive geriatric assessment (CGA) is a multidimensional approach aimed at assessing the physical, psychological, and functional capabilities of older adults to develop a coordinated and integrated care plan [10,11], which is essential for delivering services that meet the unique and diverse care needs of the elderly [25]. Research indicates that timely provision of fundamental care informed by CGA can postpone nursing home admissions, improve functional abilities, and reduce hospital service utilization [10]. Although the significance of integrating CGA into a care plan designed to support older adults in aging at home is widely recognized, there is limited research that synthesizes the various approaches and tools used for assessing the care needs of this population.

Previous research has typically focused on specific assessment domains or mainly studied the validity of individual assessment tools. For example, a systematic review by Figueiredo and colleagues (2018) examined multidimensional measures of health needs among older adults living at home [26]. The review provided a list of assessment dimensions and the corresponding instruments used. However, the primary focus was on evaluating the validation status of the identified assessment tools, without providing details on the specific measurements associated with these tools. Additionally, some studies focused exclusively on specific aspects of assessment. For instance, George et al. (2021) concentrated solely on measuring intrinsic capacity in older adults, including factors such as cognitive function, mobility, nutritional status, sensory functions, and depressive symptoms [27].

Thus, there remains a need for a comprehensive examination of the domains commonly assessed in this context, especially in home care settings. The complexity of aging necessitates an integrated approach that considers not only health-related factors but also the broader dimensions of an individual’s life, including functional, social, and environmental aspects. Understanding the major domains of assessment is essential for standardizing the assessment that aligns with the diverse needs of older individuals. This systematic review aims to present a comprehensive and standardized set of assessment domains for evaluating the health and functional status of older adults receiving home-based aged care services, thereby addressing limitations in existing assessment methods and providing the detailed information necessary to develop personalized care plans.

## 2. Materials and Methods

### 2.1. Study Design

This systematic review followed a structured methodology to identify and evaluate existing literature on assessing older people for home care services. It aimed to identify, analyze, and synthesize key domains of various assessment tools and approaches, following PRISMA (Preferred Reporting Items for Systematic Reviews and Meta-Analyses) guidelines [28]. The review was not registered.

### 2.2. Eligibility Criteria

Eligibility criteria were developed using the PCC (Population/Concept/Context) framework [29]. To be included, studies had to focus on (1) older adults (Population), (2) assessments (Concept), and (3) home care settings (Context). Only original peer-reviewed articles published from 2013 onwards were considered to reflect recent trends and ensure relevance. Studies not published in English or lacking full-text availability were excluded, as was grey literature. The inclusion and exclusion criteria are presented in Table 1.

### 2.3. Data Sources and Search Strategy

A comprehensive search was conducted between September and October 2023 across multiple databases, including Medline via Ovid, PsycINFO via Ovid, CINAHL via EBSCO, Web of Science, and Scopus, to ensure broad coverage of the literature. The search strategy was developed with specialized librarians in health to ensure the inclusion of all relevant studies. The search terms included a combination of Boolean keywords and MeSH terms such as “older adult” OR “aged” OR “elderly”, “assessment” OR “need assessment” OR “geriatric assessment”, and “home care” OR “home health care” OR “home care services.” Reference lists of the included studies were also reviewed. The search strategy is described in Supplementary Table S1.

### 2.4. Data Extraction

Two researchers (W.F. and P.L.) independently reviewed the literature for inclusion and exclusion based on predefined eligibility criteria. Titles and abstracts were initially screened, and then reviews of full-text articles were carried out. To minimize bias and ensure objectivity, each reviewer applied the criteria without knowing the other’s decisions. The reviewers discussed each case of disagreement to reach a consensus. In cases where consensus could not be reached, the research team held a further discussion to make a final decision.

Data extraction was performed using a standardized form, capturing (1) study characteristics (e.g., authors, year of publication, country, study design, and sample size), (2) sample characteristics (e.g., age, characteristics, and care setting), (3) assessments (e.g., assessment tools, assessment domains, and measurements), and (4) key findings. The data extraction sheet is presented in Supplementary Table S2.

### 2.5. Quality Assessment

Two researchers (W.F. and P.L.) independently assessed the quality of each study using the Mixed Methods Appraisal Tool (MMAT), version 2018 [30], a comprehensive instrument designed to assess the methodological quality of studies across various designs, including quantitative, qualitative, and mixed methods. The MMAT includes five study designs, each with a distinct set of five methodological quality criteria. For each criterion, responses can be classified as “yes”, “no”, or “cannot tell”, with “yes” assigned a value of 1 and “no” assigned a value of 0. Each study was appraised based on its research design, and we adopted the average results from the two raters. After appraising the individual studies, we synthesized the results by categorizing the studies into three quality levels: high, moderate, and low. Specifically, studies that met all five “yes” responses across all criteria were considered high quality, those with 3–4 “yes” responses were considered moderate quality, and studies with two or fewer “yes” responses were considered low quality. The average MMAT results are summarized in Supplementary Table S3.

### 2.6. Data Synthesis

This review used the WHO ICF classification framework for data synthesis [31]. The ICF framework provides an international standard that incorporates physical, mental, social, and environmental factors for defining and evaluating an individual’s health and functioning. The framework encompasses four main categories, including body functions, activities and participation, environmental factors, and body structure [31]. This review applied the ICF framework to systematically categorize major assessment domains for the care of older adults, aiming to improve standardization and consistency in assessment and facilitate a comprehensive approach to addressing diverse care needs.

A deductive synthesis approach was employed to summarize and integrate the findings from the included studies. The data synthesis focused on comparing the different assessment tools and highlighting the major domains assessed for older people in home care settings.

## 3. Results

### 3.1. Overview of Included Studies

**Figure 1:**
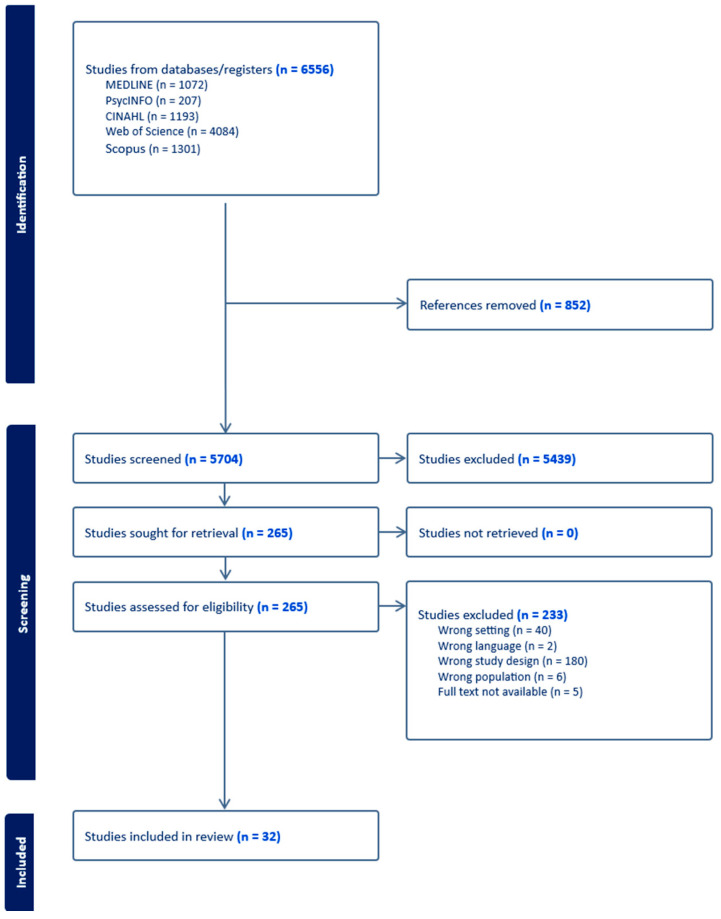
PRISMA flow diagram of literature search and study selection.

The database searches initially yielded a total of 6556 records after removing duplicates. Upon initial screening of titles and abstracts, 265 articles were identified for further full-text review. A total of 32 studies met the inclusion criteria and were included in this systematic review. The studies varied in design, including observational studies (such as cross-sectional studies, case-control studies, and longitudinal studies), randomized controlled trials (RCTs), exploratory studies, and qualitative studies. The sample sizes ranged from 25 to 126,423 participants, with most studies focusing on older adults aged 65 and above. The studies were conducted across diverse geographical locations, including Europe, North America, Asia, and Australia. Most studies were conducted in developed countries, with Norway (*n* = 5), the US (*n* = 4), and Canada (*n* = 3) leading the contributions. The PRISMA flow diagram of the literature search and study selection is shown in Figure 1.

### 3.2. Assessment Tools and Domains Identified by ICF Categories

The review identified various assessment tools used in home care settings, categorized according to the WHO ICF framework. These tools and domains were grouped into three main categories: (1) body functions, (2) activities and participation, and (3) environmental factors. Our review did not discuss the ICF category of body structure, as it pertains to specific medical evaluations (e.g., organ functions) typically performed by doctors or geriatric specialists in clinical settings. Instead, our review focused on general assessments carried out by qualified health professionals, such as nurses, occupational therapists, or physical therapists, in home care settings. Two researchers were involved in mapping ICF codes to assessment tools. We first identified the relevant tools or instruments. Then, we conducted a thorough analysis to identify the specific domains each tool incorporates, before assigning the ICF code that most accurately represents the domain assessed.

#### 3.2.1. Body Functions

This category includes tools and domains that access the physiological and psychological (mental) functioning of older adults (see Table 2).

- Physical Functions

Nine studies (28.1%) assessed physical functions, focusing on muscle power (e.g., handgrip and leg strength) [7,24,32,33,34,35], muscle endurance (e.g., leg endurance) [7,18,32,34,36], and movement functions (e.g., gait speed) [34,35,36]. Physical functions were commonly assessed with performance-based measures, including measurements like handgrip strength (HGS) [7,24,32,33,34,35], the Chair Stand Test (CST) [7,18,32,34,36], and gait speed (GS) [34,35,36]. In addition, instruments such as the Fried Frailty Phenotype (FFP) [32,34,37] and the Short Physical Performance Battery (SPPB) [33,34] also assess some of these functions.

- Mental Functions

A total of 24 studies (75.0%) assessed mental functions including three main assessment domains: cognitive functions, emotional functions, and intellectual functions.

1. Cognitive Functions

A total of 22 studies (68.8%) assessed cognitive functions [4,5,7,8,10,11,17,18,19,24,25,32,35,38,39,40,41,42,43,44,45,46]. We identified the five most assessed components of cognitive functions, including memory [4,17,18,19,35,38,40,41,42,43,44,45,46], language [4,17,18,19,35,40,41,42,43,44,45,46], orientation [4,17,18,35,38,40,41,44,45], attention [4,17,18,24,35,40,41,44,45], and calculation [4,17,18,35,40,44].

Cognitive functions were primarily assessed using tools like the Mini Mental State Examination (MMSE) [4,17,18,32,35,40,44], Cognitive Performance Scale (CPS) [19,42,43,46], Informant Questionnaire for Cognitive Disorders in the Elderly (IQCODE) [4,40,44], Hodkinson Abbreviated Mental Test (AMT) [9,10,39], and Montreal Cognitive Assessment (MoCA) [41,45]. Measurements such as the Clock Drawing Test (CDT) [4,44], Mini-Cog (MC) [19], and Trail Making Test (TMT) [24] were also commonly used for assessing cognitive functions. These tools are crucial for detecting early signs of cognitive impairments, enabling timely interventions and appropriate care and support.

2. Emotional Functions

A total of 22 studies (68.8%) assessed emotional functions such as depressive symptoms [4,5,8,9,10,11,13,17,18,19,25,32,34,35,38,40,41,42,44,45,46,47]. The most frequently used assessment tools were the Geriatric Depression Scale (GDS) or its short-form version, GDS-SF [9,10,17,18,32]; the Center for Epidemiological Studies—Depression Scale (CES-D) [34,35,41,47]; the Depression Rating Scale (DRS) [25,42,46]; and the Cornell Scale for Depression in Dementia (CSDD) [40,44]. These instruments measured common indicators of depression such as mood, sleep functions, satisfaction with life, sense of loneliness, and feelings of sadness.

3. Intellectual Functions

Three studies (9.4%) assessed intellectual functions such as the severity of symptoms of dementia [4,38,44], which was primarily quantified by the Clinical Dementia Rating Scale (CDR) [4,38,44].

- Digestive Functions

Nutritional status was assessed in 18 studies (56.3%) [5,7,8,9,11,18,25,32,33,34,35,37,38,39,45,48,49,50], using tools like the Mini Nutritional Assessment (MNA) [7,9,18,32,33,48,50] and the Mini Nutritional Assessment—Short Form (MNA-SF) [45,49,50]. Measurements such as digestive functions, appetite, chewing or swallowing difficulties, weight loss, and BMI were commonly assessed items to determine the risk of malnutrition in older adults.

- Other Body Functions

Sensory functions such as seeing and hearing were commonly assessed [19,25,38,42,45,47], but the measurements were not specified. Some studies also conducted fall risk assessment [5,38,39,45] and pain measurement [5,42,45] using tools like the Falls Risk Assessment Tool (FRAT) [39] and the Pain Scale [42], respectively.

**Table 2:** Major assessment tools and domains in the ICF category of body functions.

Assessment Aspects	Description	Assessment Domain	ICF Code	Example Tools/Measures	References
Physical functions	Assessment of muscle functions, including muscle power, muscle endurance, and movement functions	Handgrip strength Leg endurance Standing balance Walking speed	Muscle Functions b730 Muscle power functions b740 Muscle endurance functions Movement Functions b770 Gait pattern functions	HGS CST GS FFP SPPB	[7,24,32,33,34,35] [7,18,32,34,36] [34,35,36] [32,34,37] [33,34]
Mental functions	Assessment of cognitive functions, emotional functions, and intellectual functions	Cognitive Functions Memory Language Orientation Attention Calculation Abstraction Judgement Decision-making Problem-solving	Global Mental Functions b114 Orientation functions Specific Mental Functions b140 Attention functions b144 Memory functions b164 Higher-level cognitive functions b1640 Abstraction b1641 Organization and planning b1642 Time management b1645 Judgement b1646 Problem solving b167 Mental functions of language b172 Calculation functions	MMSE CPS IQCODE AMT MoCA CDT MC TMT	[4,17,18,32,35,40,44] [19,42,43,46] [4,40,44] [9,10,39] [41,45] [4,44] [19] [24]
		Emotional Functions Depressive symptoms Mood Sleep functions Aggressive behavior	Specific Mental Functions b152 Emotional functions b134 Sleep functions b1340 Amount of sleep b1343 Quality of sleep	GDS CES-D DRS CSDD	[9,10,17,18,32] [34,35,41,47] [25,42,46] [40,44]
		Intellectual Functions Severity of dementia	Global Mental Functions b117 Intellectual functions	CDR	[4,38,44]
Digestive functions	Assessment of nutritional status	Weight loss Dehydration Appetite Food intake Eating difficulties	b510 Ingestion functions b5101 Biting b5102 Chewing b5105 Swallowing b515 Digestive functions b5152 Absorption of nutrients b530 Weight maintenance functions	MNA MNA-SF	[7,9,18,32,33,48,50] [45,49,50]
Others	Assessment of sensory functions, fall risks, and pain	Vision Hearing Fall risk Pain	b210 Seeing functions b230 Hearing functions b2402 Sensation of falling b280 Sensation of pain	- - FRAT PS	- - [39] [42]

AMT: Hodkinson Abbreviated Mental Test; CDR: Clinical Dementia Rating Scale; CDT: Clock Drawing Test; CES-D: Center for Epidemiological Studies—Depression Scale; CPS: Cognitive Performance Scale; CSDD: Cornell Scale for Depression in Dementia; CST: Chair Stand Test; DRS: Depression Rating Scale; FFP: Fried Frailty Phenotype; FRAT: Falls Risk Assessment Tool; GDS: Geriatric Depression Scale; GS: gait speed; HGS: handgrip strength; IQCODE: Informant Questionnaire for Cognitive Disorders in the Elderly; MC: Mini-Cog; MMSE: Mini Mental State Examination; MNA: Mini Nutritional Assessment; MNA-SF: Mini Nutritional Assessment—Short Form; MoCA: Montreal Cognitive Assessment; PS: Pain Scale; SPPB: Short Physical Performance Battery; TMT: Trail Making Test.

#### 3.2.2. Activities and Participation

This category includes tools that assess older adults’ ability to perform daily activities and participate in life situations. Activity refers to the act of carrying out a specific task or action by an individual [31]. Participation involves being engaged in a particular life situation. Activity limitations refer to the challenges a person may encounter when performing activities, while participation restrictions relate to the difficulties an individual might face in being involved in life situations [31]. The review identified three major aspects under this category: morbidity, self-care, and domestic life (see Table 3).

- Mobility

Mobility assessments include an individual’s ability to change and maintain body position, walk, and move. The review identified the Timed Up and Go (TUG) Test as the primary performance-based measure for assessing mobility in older adults [17,24,32,33,36].

- Self-Care

Self-care abilities refer to the ability to care for oneself in home settings, indicating independence in basic activities of daily living (ADLs) [31]. Common tools included the Barthel ADL Index (B-ADL) [7,10,17,18,19,24,25,33,38,39], the Katz Index of Independence in ADLs (K-ADL) [9,13], and the ADL Self-Performance Hierarchy (P-ADL) [42]. Primary ADL tasks assessed included eating, bathing, grooming, dressing, continence, toileting, transferring, walking, and stair climbing.

- Domestic Life

Domestic life involves the ability to perform everyday tasks, often referred to as instrumental activities of daily living (IADLs) [31]. The Lawton IADL Scale (L-IADL) was most used to assess the ability of older adults to live independently at home [9,13,17,18,33,38,40,44], which focuses on functions such as the ability to use a telephone, shopping, food preparation, housekeeping (cleaning, house maintenance, and laundering), mode of transportation, responsibility for one’s own medication, and finance management.

- Others

The review also identified assessment for interpersonal interactions and relationships among older adults [9,11,13,19,43], including informal social relationships with friends, neighbors, acquaintances, co-inhabitants, and peers, as well as family relationships. Some studies also assessed economic life (e.g., financial situations) within the major life areas [10,11,17,35].

**Table 3:** Major assessment tools and domains in the ICF category of activities and participation.

Assessment Aspects	Description	Assessment Domains	ICF Code	Example Tools/Measures	References
Mobility	Assessment of ability to change and maintain body position, walk, and move	Ability to change and maintain body position, walk, and move	Changing and Maintaining Body Position d410 Changing basic body position d415 Maintaining body position d420 Transferring oneself Walking and Moving d450 Walking d455 Moving around	TUG	[17,24,32,33,36]
Self-care	Assessment of ability to care for oneself, i.e., the level of independence in performing basic ADLs	Eating, bathing, grooming, dressing, continence, toileting, transferring, walking, and stair climbing	d510 Washing oneself d520 Caring for body parts d530 Toileting d540 Dressing d550 Eating d560 Drinking	B-ADL K-ADL P-ADL	[7,10,17,18,19,24,25,33,38,39] [9,13] [7]
Domestic life	Assessment of ability to carry out domestic and everyday actions and tasks, i.e., IADLs	Ability to use a telephone, shopping, meal preparation, housework, mode of transportation, responsibility for one’s own medication, and managing finances	Household Tasks d630 Preparing meals d640 Doing housework	L-IADL	[9,13,17,18,33,38,40,44]
Others	Assessment of interpersonal interactions and relationships, and economic life	Social relationships Family relationships Financial situations	General Interpersonal Interactions Particular Interpersonal Relationships d740 Formal relationships (with professionals or service providers) d750 Informal social relationships (casual relationships) d760 Family relationships Major Life Areas d870 Economic self-sufficiency Community, Social and Civic Life d910 Community life	Self-reported assessments	[9,10,11,13,17,19,35,43]

B-ADL: Barthel ADL Index; K-ADL: Katz Index of Independence in ADLs; L-IADL: Lawton IADL Scale; P-ADL: ADL Self-Performance Hierarchy; TUG: Timed Up and Go Test.

#### 3.2.3. Environmental Factors

This category includes assessments that consider the physical and social environment surrounding older adults. The review identified “products and technology”, “support and relationships”, and “services” as the major aspects (see Table 4).

- Products and Technology

This refers to the “natural or human-made products, systems, equipment, and technologies in an individual’s immediate environment” [31]. We identified measurements such as accommodation, home environment, and living conditions [5,8,25,35,38,45]. Some studies used self-reported questionnaires, whereas instruments like the Resident Assessment Instrument for Home Care (interRAI-HC) were commonly used in some studies [5,25,45].

- Support and Relationships

This refers to the practical physical or emotional support assistance to older people in their daily life [31]. Such measurements included social networks [11,13,38], social involvement and interactions [9,19,35,43,44,45], and living arrangements [11,17,18]. These assessments were often conducted through self-reported questionnaires, whereas some studies used instruments like the Intellectual Disability Rating Scale (IDRC) for social interactions and interpersonal relationships [19], Dukes Social Support Index (DSSI) for social support [10], and Social Network Analysis (SNA) for social networks [13].

- Services

Services outline the various health, social, and other services tailored to fulfill older adults’ needs. With regards to the assessments in available services for this population, the types of formal and informal care [4,5,8,10,44,45] and the use of health and social care service [10,40] were most assessed. They were assessed through self-reported questionnaires in most of the included studies. In addition, some studies assessed the home environment and living conditions of older people [5,8,25,35,38,45].

**Table 4:** Major assessment tools and domains in the ICF category of environmental factors.

Assessment Aspects	Description	Assessment Domains	ICF Code	Example Tools/Measures	References
Products and technology	Assessment of natural or human-made products, systems, equipment, and technologies	Accommodation Home environment Living conditions	Products and Technology	interRAI-HC	[5,25,45]
Support and relationships	Assessment of practical physical or emotional support to older people in daily activities	Social networks and support Social involvement and interactions Living arrangements	Support and Relationships	IDRC DSSI SNA	[19] [10] [13]
Services	Assessment of various health, social, and other services tailored to fulfill older adults’ needs	Types of formal and informal care Use of health and social care service Home environment Living conditions	Services, Systems, and Policies e575 General social support services, systems, and policies e580 Health services, systems, and policies	Self-reported assessments	[4,5,8,10,25,35,38,40,44,45]

DSSI: Dukes Social Support Index; IDRC: Intellectual Disability Rating Scale; interRAI-HC: Resident Assessment Instrument for Home Care; SNA: social network analysis.

## 4. Discussion

This systematic review sought to elucidate which assessment domains have been put into practice for older people receiving home care services to date. Timely review of this information would facilitate the development of a standardized and inclusive assessment method in this context.

This systematic review explored the major domains of a diversity of assessment tools used for assessing older adults receiving home care services, structured based on the WHO ICF framework. By aligning the identified assessment domains with ICF categories, the review provided a comprehensive understanding of what and how various aspects of health and functioning are assessed in this population. The review identified three primary categories, including (1) body functions, (2) activities and participation, and (3) environmental factors. Body functions included physical functions and mental functions. Mobility, self-care, and domestic life were three major dimensions within the category of activities and participation. Regarding the environmental factors, support, relationships and services to older people were most assessed. Specifically, the major domains assessed in the included studies were physical functions, cognitive functions, psychological well-being, functional capacities, and nutritional status.

The review identified a variety of tools as common measurements for body functions, including physical functions and mental functions. These tools offer a comprehensive framework for assessing both physical and mental impairments, as well as for determining necessary care and support needs. Physical functions are commonly determined by older adults’ muscle strength and endurance, such as handgrip strength and gait speed. It is also essential to assess the nutritional status of older adults receiving home care services due to their increased vulnerability to malnutrition [33,50], and it is a significant factor contributing to the loss of independence as individuals age [32]. This susceptibility can arise from issues such as dementia, depression, immobility, reduced oral intake, chewing and swallowing difficulties, inability to eat independently, anorexia, and nausea or vomiting [49].

In addition, under mental functions, cognitive decline represents a significant health issue for older adults. Like other age-related deficits, such as declines in renal function, cognitive impairments often progress unnoticed in many older adults [41]. With the growing population of older adults at risk for cognitive decline, it is essential to enhance the understanding of their overall cognitive abilities [41]. Cognitive assessments are instrumental in detecting early signs of cognitive impairments in older adults, allowing for timely care and support for them [41]. Similarly, Xiang et al. [47] expected a significant prevalence of depression among older adults receiving home-based aged care, attributable to a decline in physical and social independence. In addition, depression is a common contributor of emotional distress in the older population and can also lead to diminished physical functioning and a lower quality of life [44].

Regarding additional body functions, Davidson et al. [42] demonstrated that older adults with vision and hearing impairments experience higher rates of cognitive decline and functional dependence. These individuals may face increased risks for adverse health outcomes, including challenges in performing ADLs and IADLs, as well as higher incidences of depression, communication difficulties, and social isolation, increasing the need to receive health care services in their own homes [19,38]. Assessments of visual and hearing functions are thus crucial for identifying sensory impairments that can impact the independence at home and quality of life of older adults [42].

In the category of activities and participation, the Barthel ADL Index is frequently used to assess basic ADLs such as eating, personal hygiene, and toileting. The review found that such an assessment is essential for caregivers and aged care providers to assess the extent of independence while performing basic ADLs and understand the assistance required by older adults in their daily life [39] in order to improve the quality and effectiveness of care provided [38]. In addition, the review identified the importance of the assessment of functional independence and mobility in older adults. These findings are in line with Predebon et al. [17], who underscored the importance of regular assessments in independent living skills to ensure safety in home-based care environments.

Additionally, under the category of environmental factors, the review highlighted the importance of social support and community involvement for the health and social well-being of older adults. Social support measures offer valuable insights into the scope of social networks [11,13] and community involvement [19,43,44,45] among older adults, which are essential for effective care planning. The review highlighted the positive impact of support services on the overall health of older adults and the quality of care provided to them, emphasizing the need for strategies to promote community involvement. The review also emphasized the role of an accessible and safe home environment in supporting independent living of older adults, corroborating studies that link home safety with better health outcomes [51,52].

The identified instruments provide a comprehensive collection of methods for assessing various aspects of health and functioning in older adults. Performance-based assessments were utilized to measure physical functions and mobility, whereas assessment of interpersonal relationships and services available were primarily through self-reported methods, potentially introducing biases such as social desirability and recall inaccuracies [27,53].

However, we found heterogeneity and low concordance among the included studies when measuring some of the domains. This was particularly manifest in the assessment of physical functions and nutritional status, which would make cross-study comparisons difficult. In addition, even for the same tool used, there were variations in the measuring methods and cut-off points adopted. For example, when assessing mobility using the TUG test, which measures the time needed to stand up from a chair, walk three meters, turn around, return, and sit down again, the cut-off value varied in the studies. Certain studies established a cut-off value of over 20 s to distinguish between older adults with moderate to severe limitations and those with no or slight limitations [17,33], whereas Naess et al. [24] suggested that over 30 s to perform TUG indicates a mobility problem. Similarly, the classification of nutritional status is also heterogeneous in studies using the MNA scale. Research often establishes a cut-off score of 23.5 to signify a risk of malnutrition and a score of 17 to denote malnourishment [9,18,33,50], as per the official assessment criteria of the scale. However, Berggren et al. [48] employed cut-off values of 13 and 9, while Swan et al. [7] used 11 and 7, respectively, for this classification. These heterogeneities necessitate more integrative and standardized assessment methods in this context.

Variability in the administration or scoring of assessment tools can reduce the generalizability of results. To address this, assessment tools should be culturally and linguistically adapted to ensure they are appropriate for diverse populations [54]. It is also crucial to establish clear guidelines and standard operating procedures for administering assessments in home care settings. Additionally, all individuals involved in administering assessments must be trained in these standardized procedures.

Additionally, current assessments often compartmentalize physical, cognitive, psychological, and social health and well-being, which overlooks the interconnections between different domains of health and functioning and may lead to fragmented care [27]. Moreover, most assessment tools focus on physical and cognitive aspects, often overlooking social and environmental factors, which are also important aspects highlighted by the research as vital for holistic aged care [21,22]. However, assessing environmental factors and social support is crucial for a comprehensive understanding of an individual’s health and functioning. These factors significantly influence an individual’s ability to participate in daily life and their overall quality of life [55]. When these aspects are not adequately assessed, it can result in an incomplete picture of an individual’s health and functioning. On the other hand, integrating assessments of these factors provides a more holistic and accurate view, enabling more effective interventions and better long-term health outcomes.

These findings are consistent with previous studies emphasizing the multifaceted nature of assessments for older adults and underscoring the necessity for comprehensive assessment methods [21,22]. For instance, combining physical health assessments with social support evaluations can help identify older adults at risk of isolation and its associated health risks [56]. Some studies have conducted comprehensive assessments using instruments or questionnaires such as the interRAI-HC [5,10,25,42,43,45,46], Support Needs Assessment (SNA) [10], 36-item Short Form Survey (SF-36) [10], and EASY-Care [8]. However, the administration of these assessments in home care settings is varied and inconsistent. Specially, the assessment domains across these instruments are diverse and often lack standardization, meaning no single instrument can fully address the comprehensive needs of older adults. This highlights the need for a more standardized approach. Comprehensive and standardized assessments could help streamline assessment practices, improve the accuracy of care needs identification, facilitate more personalized and inclusive aged care plans, and enhance the quality of aged care services for older people aging at home.

The findings of this review have significant implications for practice and future research of home-based aged care. Previous research has typically focused on specific health domains or mainly studied the validity of individual assessment tools. In contrast, this review examined a broad spectrum of assessment tools through the WHO ICF framework to comprehensively address the physical, mental, social, and environmental needs of older adults. A similar study by Abdi et al. (2019) also used the ICF framework to understand the care and support needs of older adults; however, it did not detail the specific methods employed to assess and identify these needs [20]. By taking this integrated approach, the study goes beyond a limited approach and proposes a multifaceted assessment framework essential for effective aged care.

However, an ethical dilemma arises when comprehensive assessment tools are used to gather information about an individual’s needs, while there is a lack of available resources to meet those needs. Ethically, it is important to collect this information to ensure that care planning is comprehensive and individualized, but this must be done transparently and with a clear understanding of which resources are available. Therefore, it is essential that assessments must be accompanied by clear communication about the constraints on the care system. If certain needs cannot be addressed, it is important to inform individuals of these constraints upfront and provide alternative solutions or support networks.

A notable strength of the study is its application of the WHO ICF framework, standardizing the naming and classification of major domains of various assessment tools identified, and using universally recognized and accepted terminology related to health and functioning. This standardization enhances understanding and communication among stakeholders involved in home care settings. Although the ICF framework is widely recognized for its strengths, owing to its comprehensive, multi-dimensional approach to evaluating health and functioning, it is not without criticism. Potential concerns include its complexity, cultural sensitivity, and feasibility in resource-constraint settings [57]. In some contexts, adaptations to the framework could be beneficial.

The study also has some limitations. First, variations in how specific domains are measured across different assessment tools may limit the comparability of results between studies, potentially affecting the generalizability of the findings. Also, the review was limited to identified studies through specific databases and a defined publication period (2013 onward), which may have led to the exclusion of pertinent data and introduced selection bias. The exclusion of non-English studies and grey literature may also limit the generalizability of the findings.

Future research can focus on exploring integrative and standardized assessments that encompass the major ICF categories and relevant domains. Standardized assessments are crucial not only for improving personalized care but also for influencing policy development and service delivery across broader systems. They are particularly valuable for comparing service provision across various units, identifying regional disparities, and monitoring trends over time [58]. They can provide policymakers with evidence-based insights that can shape resource allocation, improve service quality, and prioritize areas that need improvement, making them essential for the development of more equitable and effective aged care services.

Validity and reliability are critical attributes for assessment instruments [27,28]. For example, when assessing cognitive functions, the concurrent use of validated instruments or questionnaires that measure subjective functions (e.g., memory) may reduce the likelihood of ceiling effects or educational bias [27,53]. The consistency and accuracy of composite functioning scores can be enhanced by employing measurement instruments that are less susceptible to bias [27]. Encouraging research on the validation of assessment tools for culture-specific contexts will contribute to their applicability and generalizability for different cultural contexts. In addition, longitudinal studies or RCTs are needed to examine the long-term impact of these instruments on care needs identification and enable necessary adaption to optimize care strategies and improve older adults’ health and well-being.

Our findings may have some policy implications. To enhance home care services, national guidelines may consider standardizing assessment tools and practices, adopting the WHO ICF framework for consistency. Integrative assessments can enhance aged care in home care settings by focusing on practical, patient-centered applications. It is essential that health professionals are well-trained in administering these assessments. Training programs for care providers and informal caregivers on these standardized tools will improve the accuracy and reliability of assessments, ensuring care plans are effectively tailored to older adults’ needs. Additionally, investing in digital health technologies, such as mobile applications and telehealth services, will facilitate comprehensive assessments and real-time monitoring of older adults’ health and well-being. Strengthening community- and home-based support systems is crucial, with the establishment of community centers offering health, social, and recreational services. Formulating policies that integrate home care services into the broader healthcare system, ensuring adequate funding, resources, and regulatory oversight, will maintain high standards of care. Regular evaluations, coupled with personalized interventions, help ensure that the physical, emotional, and social needs of older adults are effectively met in the home environment.

## 5. Conclusions

In summary, this systematic review synthesized a collective of assessment tools and the major domains assessed for older adults in home care settings. By categorizing these domains under the ICF categories, the review provides a structured approach to understanding the multifaceted aspects of the health and functioning of older adults. The review highlights the compartmentalization of existing assessment approaches. Therefore, future research should aim to explore more integrative methods that address the interconnected nature of health and well-being in older adults. Continuing research and refinement of assessment methods are crucial to ensure optimal assessment results. This will, in turn, help aged care providers grasp the health and functioning status of older adults and monitor changes, ensuring that older adults receive tailored care and support to improve their quality of life when aging in their own homes.

## Figures and Tables

**Table 1:** Inclusion and exclusion criteria based on the PCC framework.

PCC Framework	Inclusion Criteria	Exclusion Criteria
Population Older people	Studies were included if they: Focus on older people	Focus exclusively on: Other age groups OR Caregivers OR Healthcare professionals OR Any other groups
Concept Assessment	Studies were included if they: Describe assessment tools or assessment domains for older people, including physical, mental, cognitive, social, and environmental aspects	Focus exclusively on: Development, reliability, and validity studies of assessment tools OR Feasibility, quality, or effectiveness of assessment tools OR Outcomes or effects of assessments OR Quality of healthcare services or quality of life of aged care service consumers OR Prevalence of healthcare needs or unmet needs OR Incidence of certain health conditions or symptoms
Context Home care setting	Studies were included if they: Describe assessments taking place in home care settings	Focus exclusively on: Assessments in clinical or institutional settings, such as assessments taking place in hospitals, clinics, community healthcare centers, nursing homes, etc.

## Data Availability

Data are contained within the article or Supplementary Materials. The original contributions presented in the study are included in the article/Supplementary Materials, and further inquiries can be directed to the corresponding author.

## Supplementary Materials

The following supporting information can be downloaded at: https://www.mdpi.com/article/10.3390/healthcare12232468/s1, Supplementary Table S1: Search strategy; Supplementary Table S2: Data extraction sheet; Supplementary Table S3: Average MMAT results.
