# Supplementary material for: The Major Domains of Comprehensive Assessment Tools for Older Adults Requiring Home-Based Aged Care Services: A Systematic Review
Source: Healthcare (Basel). 2024 Dec 6;12(23):2468. doi: 10.3390/healthcare12232468 (PMC11641082; doi:10.3390/healthcare12232468)
Supplement: Supplementary file 1 [file healthcare-12-02468-s001.zip › healthcare-3306920-supplementary.pdf]

Supplementary Materials

Supplementary Table S1. Search strategy

No.	Databases	Search strategies	No. of studies
**1**	Medline (via Ovid)	1. home care.mp. or exp Home Care Services/	
		2. (home adj3 care).mp. [mp=title, book title, abstract, original title, name of substance word, subject heading word, floating sub-heading word, keyword heading word, organism supplementary concept word, protocol supplementary concept word, rare disease supplementary concept word, unique identifier, synonyms, population supplementary concept word, anatomy supplementary concept word]	
		3. aged.mp. or exp Health Services for the Aged/ or exp Aged/	
		4. (old$2 or eld$4 or aged).mp. [mp=title, book title, abstract, original title, name of substance word, subject heading word, floating sub-heading word, keyword heading word, organism supplementary concept word, protocol supplementary concept word, rare disease supplementary concept word, unique identifier, synonyms, population supplementary concept word, anatomy supplementary concept word]	
		5. needs assessment.mp. or exp "Health Services Needs and Demand"/ or exp Needs Assessment/	
		6. (need* adj3 assess*).mp. [mp=title, book title, abstract, original title, name of substance word, subject heading word, floating sub-heading word, keyword heading word, organism supplementary concept word, protocol supplementary concept word, rare disease supplementary concept word, unique identifier, synonyms, population supplementary concept word, anatomy supplementary concept word]	
		7. geriatric assessment.mp. or exp Geriatric Assessment/	
		8. 1 or 2	
		9. 3 or 4	
		10. 5 or 6 or 7	
		11. 8 and 9 and 10	
		12. limit 11 to (english language and humans)	
		13. limit 12 to yr="2013 -Current"	1072
**2**	PsycINFO (via Ovid)	1. home care.mp. or exp Home Care/	
		2. (home adj3 care).mp. [mp=title, abstract, heading word, table of contents, key concepts, original title, tests & measures, mesh word]	

		3. exp Older Adulthood/ or exp Elder Care/ or aged.mp.	
		4. (old$2 or eld$4 or aged).mp. [mp=title, abstract, heading word, table of contents, key concepts, original title, tests & measures, mesh word]	
		5. needs assessment.mp. or exp Needs Assessment/	
		6. (need* adj3 assess*).mp. [mp=title, abstract, heading word, table of contents, key concepts, original title, tests & measures, mesh word]	
		7. geriatric assessment.mp. or exp Geriatric Assessment/	
		8. 1 or 2	
		9. 3 or 4	
		10. 5 or 6 or 7	
		11. 8 and 9 and 10	
		12. limit 11 to (human and english language)	
		13. limit 12 to yr="2013 -Current"	207
**3**	CINAHL (via EBSCO)	1. (MH "Home Health Care+") OR "home care" OR (MH "Health Services Needs and Demands+")	
		2. home N/3 care	
		3. (MH "Aged+") OR "aged"	
		4. old* or eld* or aged	
		5. (MH "Geriatric Assessment+") OR "geriatric assessment" OR (MH "Needs Assessment+")	
		6. need* N3 assess*	
		7. 1 OR 2	
		8. 3 OR 4	
		9. 5 OR 6	
		10. 7 AND 8 AND 9	
		11. Narrow by language: - english	
		12. Limiters – Published Date: - 20130101-20231231	1193
**4**	Web of Science	1. home NEAR/3 care	
		2. old* OR elder* OR aged	
		3. assess*	
		4. 2013 onwards, english	4084

**5**	Scopus	(TITLE-ABS-KEY (home W/3 care) AND TITLE-ABS-KEY (old! OR eld! OR aged) AND TITLE-ABS-KEY (“geriatric assessment” OR “need* assessment” OR assess!) AND NOT TITLE-ABS-KEY (“nursing home” OR resident! OR hospital* OR institution!)) AND PUBYEAR > 2012 AND PUBYEAR < 2024 AND (LIMIT-TO (LANGUAGE, “English”))	1301
**Total**			6556

Supplementary Table S2. Data extraction sheet

No	Study	Authors	Publication year	Country	Study design	Sample size	Age group	Sample characteristics	Study focus	Assessment instruments	Assessment domains	Measurements	Main findings
1	The relationship between frailty, functional dependence, and healthcare needs among community-dwelling people with moderate to severe dementia	Abreu, W., Tolson, D., Jackson, G. A., Staines, H., & Costa, N.	2019	Portugal	cross-sectional survey design	83	50-95 years	community-dwelling older adults diagnosed with moderate or severe dementia	1) assessment of the functional dependency and the living skills of the persons with moderate and severe dementia;<br>2) identification the unmet needs of the persons with moderate and severe dementia;<br>3) investigated whether unmet needs are linked to frailty levels	1) Clinical Dementia Rating (CDR)<br>2) Barthel ADL Index (B-ADL)<br>3) Lawton IADL Scale (L-IADL)<br>4) Edmonton Frail Scale (EFS)<br>5) Care Needs Assessment Pack for Dementia (Carenap D)	1) health conditions<br>2) psychosocial well-being<br>3) cognition<br>4) functional status<br>5) nutritional status<br>6) social support<br>7) environmental assessment<br>8) health behaviour	**1) health conditions:** vision and hearing, continence, breathing, general health status, medications, skin condition;<br>**2) psychosocial well-being:** mood, usual sleep, emotion, talking about feelings;<br>**3) cognition:** communication and interaction, memory, confusion, recognizing familiar people, making decisions and plans; severity of symptoms of dementia;<br>**4) functional status:** mobility, falls, taking part in activities, eating and drinking, looking after home, food preparation, asking for social support, toilet use, money use;<br>**5) nutritional status**<br>**6) social support**<br>**7) environmental assessment:** accommodation;<br>**8) health behaviour:** daytime activities	1) a collection of 26 healthcare needs was defined to facilitate the assessment;<br><br>2) The most common healthcare needs identified in the sample included food preparation, medication management, home care, toilet usage, sensory issues, communication and interaction, bladder and bowel concerns, eating and drinking, memory, sleep, and fall prevention;<br><br>3) even with well-organized home-care programs for individuals with dementia, there are still unmet health needs. Conducting timely healthcare assessments can help professionals prevent fragmented care and create tailored, high-quality interventions that also consider the emotional

No	Study	Authors	Publication year	Country	Study design	Sample size	Age group	Sample characteristics	Study focus	Assessment instruments	Assessment domains	Measurements	Main findings
													and psychological well-being of caregivers
2	Prevalence of frailty-related risk factors in older adults seen by community nurses	Ballard, J., Mooney, M., & Dempsey, O.	2013	Ireland	Retrospective cross-sectional clinical audit	120	>65 years	a cohort of older adults visited by community nurses in Dublin, Ireland	description the frequency of suspected cognitive impairment, risk of malnutrition, falling, and dependence in ADL	1) Hodkinson¡¯s Abbreviated Mental Test (AMT) 2) Nutritional Screening Initiative (NSI) 3) modified Barthel ADL Index 4) Falls Risk Assessment Tool (FRAT)	1) cognition 2) functional status 3) nutritional status	**1) cognition:** memory and cognitive ability; **2) functional status:** independence vs. dependency on others for ADLs, fall risk; **3) nutritional status:** risk for malnutrition	1) dependency in daily activities, which is a result of frailty, is closely linked to a lower chance of living alone and a higher likelihood of seeking community care services; 2) additional research is needed to explore how incorporating frailty screening into the referral process could improve the

No	Study	Authors	Publication year	Country	Study design	Sample size	Age group	Sample characteristics	Study focus	Assessment instruments	Assessment domains	Measurements	Main findings
													identification of community care needs among older adults
3	Muscle strength is associated with physical function in community-dwelling older adults receiving home care. A cross-sectional study	Bardstu, H. B., Andersen, V., Fimland, M. S., Raastad, T., & Saeterbakken, A. H.	2022	Norway	exploratory cross-sectional analysis	107	oldest old (>80 years)	oldest old adults who were community-dwelling and received home care due to functional and/or medical disabilities	examination of the association between maximal- and explosive muscle strength with physical function	1) Five times sit-to-stand (5TSTS)<br>2) Timed 8-feet-up-and-go (TUG-8ft) Test<br>3) Gait Speed	functional status	**functional status:** mobility, muscle endurance, walking speed	higher maximal- and explosive muscle strength was associated with better physical function in older adults receiving home care

No	Study	Authors	Publication year	Country	Study design	Sample size	Age group	Sample characteristics	Study focus	Assessment instruments	Assessment domains	Measurements	Main findings
4	Symptom burden in patients with home care who are at risk for malnutrition: A cross-sectional study	Berggren, E., Strang, P., Orrevall, Y., Odlund Olin, A., & Tornkvist, L.	2020	Sweden	cross-sectional study	121	oldest old (>80 years)	oldest old adults who were in early palliative stages of illness	examination of the symptom burden and nutritional problems	1) Mini Nutritional Assessment (MNA)<br>2) Functional Assessment of Anorexia/Cachexia Therapy (FAACT)<br>3) Patient-Generated Subjective Global Assessment Short Form (PG-SGA)<br>4) Edmonton Symptom Assessment System (ESAS)	nutritional status	**nutritional status:** malnutrition risk, food intake, eating difficulties, appetite, nausea	along with performing structured screenings for malnutrition, healthcare professionals should recognize that the risk of malnutrition often comes with challenges related to food and eating, as well as adverse physical, psychological, and social effects stemming from eating difficulties

No	Study	Authors	Publication year	Country	Study design	Sample size	Age group	Sample characteristics	Study focus	Assessment instruments	Assessment domains	Measurements	Main findings
5	Unmet needs of community-residing persons with dementia and their informal caregivers: Findings from the maximizing independence at home study	Black, B. S., Johnston, D., Rabins, P. V., Morrison, A., Lyketsos, C., & Samus, Q. M.	2013	US	cross-sectional study	246	oldest old (>80 years)	oldest old adults who were community-residing persons with dementia (PWD)	in-home assessments of dementia-related needs, determining the prevalence and correlates of unmet needs	1) Johns Hopkins Dementia Care Needs Assessment<br>2) Mini Mental State Examination (MMSE)<br>3) Neuropsychiatric Inventory Questionnaire (NIQ)<br>4) Cornell Scale for Depression in Dementia (CSDD)<br>5) Zarit Burden Inventory (ZBI)<br>6) Geriatric Depression Scale (GDS)<br>7) Informant Questionnaire for Cognitive Disorders in the Elderly (IQCODE)<br>8) 12-item Short Form Survey (SF-12)<br>9) Lawton-Brody IADL Scale (L-IADL)<br>10) Caregiver Activity Survey	1) health conditions<br>2) psychosocial well-being<br>3) cognition<br>4) functional status<br>5) social support<br>6) caregiver-related<br>7) quality of life<br>8) care needs	**1) health conditions:** reviews of medical health histories, medications, physical health problems;<br>**2) psychosocial well-being:** reviews of mental health histories, mental status _ depressive symptoms, neurological examinations;<br>**3) cognition:** severity of dementia, cognitive disorder;<br>**4) functional status**<br>**5) social support:** use of health and social services;<br>**6) caregiver-related:** caregiver self-rated health;<br>**7) quality of life**<br>**8) care needs:** participant and caregiver dementia-related needs	unmet needs may be more pronounced among residents of minority and low-income communities, caregivers with lower educational attainment, and individuals in the early stages of dementia

No	Study	Authors	Publication year	Country	Study design	Sample size	Age group	Sample characteristics	Study focus	Assessment instruments	Assessment domains	Measurements	Main findings
6	Cognitive function and its risk factors among older us adults living at home	Dale, W., Kotwal, A. A., Shega, J. W., Schumm, L. P., Kern, D. W., Pinto, J. M., Pudelek, K. M., Waite, L. J., & McClintock, M. K.	2018	US	household survey	3129	62-90 years	not specified	examination of the association between MoCA-SA scores and determined the sociodemographic and health-related risk factors	1) survey-adapted Montreal Cognitive Assessment (MoCA-SA)<br>2) Centre for Epidemiological Studies-Depression (CES-D) scale<br>3) Assessment of ADLs and IADLs, instrument not specified	1) psychosocial well-being<br>2) cognition<br>3) functional status:<br>4) health behaviours	**1) psychosocial well-being:** depressive symptoms;<br>**2) cognition:** orientation, naming, executive function, visuo-construction, memory, attention, language, abstraction;<br>**3) functional status:** self-report of needing in 5 ADLs (using the toilet, bathing, dressing, eating, and transferring) and 7 IADLs (preparing meals, taking medications, managing finances, shopping for groceries, light housework, driving a car during the day, and using a telephone);<br>**4) health behaviours:** tobacco use, alcohol intake, and physical activity	1) MoCA-SA scores declined with increasing age, and there were significant variations based on sex, education level, and racial/ethnic groups;<br><br>2) lower cognitive performance was linked to poor physical health, functional status, and depression, whereas current health behaviours were not associated with it
7	Older adults with a combination of vision and hearing impairment experience higher rates of cognitive impairment, functional dependence, and worse outcomes across a set of quality indicators	Davidson, J. G. S., & Guthrie, D. M.	2019	Canada	secondary analysis of data collected using the RAI-HC	352656	>65 years	home care clients with both vision and hearing loss (or dual sensory impairment [DSI])	examination of hearing and vision impairments	1) Resident Assessment Instrument for Home Care (interRAI-HC)<br>2) ADL Self-Performance Hierarchy (ADL-SHS)<br>3) IADL Involvement Scale (IADL-I)<br>4) Cognitive Performance Scale (CPS)<br>5) Depression Rating Scale (DRS)<br>6) Pain Scale<br>7) Changes in Health, End-Stage Disease and Symptoms and Signs (CHESS)<br>8) Deafblind Severity Index (DbSI)	1) health conditions<br>2) psychosocial well-being<br>3) cognition<br>4) functional status<br>5) caregiver-related	**1) health conditions:** vision and hearing, frequency of pain, chronic conditions;<br>**2) psychosocial well-being:** depressive symptoms;<br>**3) cognition:** short term memory, cognitive skills for daily decision making, expressive communication;<br>**4) functional status:** level of independence in eating; functional ability in ADLs_ early (personal hygiene), middle (locomotion and toileting), and late loss (eating), 7 IADL items (housework, meal preparation, managing finances and medications, phone use, shopping, and transportation;<br>**5) caregiver-related:** distress of caregivers	1) older adults with significant hearing loss and any visual impairment exhibited the highest rates of QI;<br><br>2) individuals with DSI experience higher rates of adverse events across many health-related outcomes and QIs

No	Study	Authors	Publication year	Country	Study design	Sample size	Age group	Sample characteristics	Study focus	Assessment instruments	Assessment domains	Measurements	Main findings
8	Nutritional status in older people - An explorative analysis	Engelheart, S., Andrén, D., Repsilber, D., Forslund, H. B., & Brummer, R. J.	2021	Sweden	longitudinal study	69	>65 years	older adults who were enrolled at home health care	assessment of a broad set of nutritional status indicators in the participants	1) Mini Nutritional Assessment (MNA)<br>2) Geriatric Depression Scale (GDS)<br>3) Mini Mental Statement Examination (MMSE)<br>4) Handgrip Strength<br>5) Chair Stand Test (CST)<br>6) Timed Up and Go (TUG) test<br>7) International Physical Activity Questionnaire modified for the elderly (IPAQ-E)<br>8) Fr€andineGrimby Scale of physical activity<br>9) Pharmacotherapeutic Symptom Evaluation in 20 questions (Phase-20)<br>10) EQ-5D<br>11) Fried Frailty Phenotype (FFP)<br>12) ADL Taxonomy	1) health conditions<br>2) psychosocial well-being<br>3) cognition<br>4) functional status<br>5) nutritional status<br>6) health behaviour<br>7) quality of life	**1) health conditions:** sensory functions (vision and hearing);<br>**2) psychosocial well-being:** depressive symptoms;<br>**3) cognition:** cognitive function;<br>**4) functional status:** frailty, iADL (cleaning, cooking, shopping, transportation and washing) and pADL (communication and grooming);<br>**5) nutritional status:** body weight change, food intake level, dehydration, appetite;<br>**6) health behaviour:** level of physical activities;<br>**7) quality of life**	there were statistically significant differences in the prevalence of malnutrition, frailty, sarcopenia, and dehydration—each representing distinct forms of inadequate nutritional status—based on individuals' component scores

No	Study	Authors	Publication year	Country	Study design	Sample size	Age group	Sample characteristics	Study focus	Assessment instruments	Assessment domains	Measurements	Main findings
9	Social and functional health of home care clients with different levels of cognitive impairments.	Garms-Homolova, V., Notthoff, N., Declercq, A., van der Roest, H. G., Onder, G., Jonsson, P., & van Hout, H.	2017	Europe	longitudinal study	2884	>65 years	individuals who received the services for at least 14 days; mildly and moderately impaired older adults	1) assessment of the ability to manage one's life with some degree of independence, to fulfill basic obligations, and to participate in social activities;<br>2) testing of the association between participants' capacity and performance in three LADLs and their cognitive performance and specific memory problems	1) Resident Assessment Instrument for Home Care (interRAI-HC)<br>2) Cognitive Performance Scale (CPS)<br>3) Assessment of IADLs, instrument not specified	1) health conditions<br>2) cognition<br>3) functional status<br>4) social support	**1) health conditions**<br>**2) cognition:** capabilities for decision-making, short-term memory, selected communication skills;<br>**3) functional status:** ability to eat independently; IADLs _ capability for coping _ managing of finances, managing of medication, and utilization of the phone over the course of last three days<br>**4) social support:** fulfillment of social obligations _ a) participation in social activities of longstanding interest; b) visits with longstanding social relations of family; c) communication on the phone or email interaction with friends or relatives	1) all clients, especially those with mild to moderate cognitive impairment, primarily engage with their close family and friends;<br><br>2) mild to moderate cognitive limitations do not prevent clients from managing routine tasks with a degree of independence

No	Study	Authors	Publication year	Country	Study design	Sample size	Age group	Sample characteristics	Study focus	Assessment instruments	Assessment domains	Measurements	Main findings
10	Toileting difficulties in older people with and without dementia receiving formal in-home care-A longitudinal study	Grimsland, F., Seim, A., Borza, T., & Helvik, A. S.	2019	Norway	longitudinal observational study	1001	≥ 70 years	older adults with and without dementia receiving formal in-home care	estimation of the prevalence of toileting difficulties	1) Lawton and Brody's Physical Self-Maintenance Scale (P-ADL)<br>2) Mini Mental State Examination (MMSE)<br>3) Clock Drawing (CDT)<br>4) Informant Questionnaire on Cognitive Decline in the Elderly (IQ-CODE)<br>5) Clinical Dementia Rating Scale (CDR)<br>6) Clock Drawing Test (CDT)<br>7) General Medical Health Rating (GMHR) scale	1) health conditions<br>2) psychosocial well-being<br>3) cognition<br>4) functional status<br>5) social support	**1) health conditions:** physical comorbidity, medications;<br>**2) psychosocial well-being**<br>**3) cognition:** severity of dementia, cognitive functions over the past 10 years;<br>**4) functional status:** toileting difficulties;<br>**5) social support:** type of formal care, receiving formal in-home care, including domiciliary care, in-home nursing care and “other types of support”	toileting difficulties were more prevalent in people with than without dementia

No	Study	Authors	Publication year	Country	Study design	Sample size	Age group	Sample characteristics	Study focus	Assessment instruments	Assessment domains	Measurements	Main findings
11	The course of depressive symptoms with decline in cognitive function - a longitudinal study of older adults receiving in-home care at baseline	Helvik, A. S., Barca, M. L., Bergh, S., Saltyte-Benth, J., Kirkevold, O., & Borza, T.	2019	Norway	longitudinal study	1001	≥ 70 years	older adults receiving in-home care	1) description of the prevalence, incidence and persistence of depressive symptoms;<br>2) exploration of the association between cognitive function and the course of depressive symptoms over time	1) Cornell Scale for Depression in Dementia (CSDD)<br>2) Clinical Dementia Rating (CDR) Scale<br>3) Lawton and Brody's Physical Self-Maintenance Scale (P-ADL)<br>4) Lawton-Brody IADL Scale (L-IADL)<br>5) Mini Mental State Examination (MMSE)<br>6) Clock Drawing Test (CDT)<br>7) Informant Questionnaire on Cognitive Decline in Elderly (IQCODE)<br>8) Neuropsychiatric Inventory (NPI)<br>9) General Medical Health Rating Scale (GMHR)	1) health conditions<br>2) psychosocial well-being<br>3) cognition<br>4) functional status<br>5) social support	**1) health conditions:** physical comorbidity, medical conditions and the use of medications;<br>**2) psychosocial well-being:** depression, anxiety;<br>**3) cognition:** memory, orientation, judgement and problem solving; neuropsychiatric symptoms _ delusion, hallucination, euphoria, agitation/aggression, disinhibition, irritability/lability, dysphoria, apathy/indifference, and aberrant motor behaviour;<br>**4) functional status:** ADLs and IADLs;<br>**5) social support:** formal level of care, community affairs, home functions, and personal care	nurses and clinicians need to consider cognitive status when assessing or observing depression in older adults who are receiving in-home care

No	Study	Authors	Publication year	Country	Study design	Sample size	Age group	Sample characteristics	Study focus	Assessment instruments	Assessment domains	Measurements	Main findings
12	Malnutrition is related to functional impairment in older adults receiving home care	Kiesswetter, E., Pohlhausen, S., Uhlig, K., Diekmann, R., Lesser, S., Heseker, H., Stehle, P., Sieber, C. C., & Volkert, D.	2013	Germany	cross-sectional study	296	>65 years	home care clients	1) examination of the association between nutritional status and functional capacity;<br>2) investigation of the impact of different MNA subscales on this association	1) Mini Nutritional Assessment (MNA)<br>2) Barthel ADL Index (B-ADL)<br>3) Lawton IADL Scale (L-IADL)<br>4) Handgrip Strength<br>5) Timed Up and Go (TUG) Test<br>6) Short Physical Performance Battery (SPPB)	1) functional status<br>3) nutritional status	**1) functional status:** mobility, functional status _ a) ADLs and IADLs; b) performance tests _ handgrip strength, leg endurance, standing balance, walking speed<br>**2) nutritional status**	functional status declined markedly from the well-nourished group to the malnourished group across all functional assessments
13	Frailty assessment of older adults, first-time applicants of public home care service in Norway	Laukli, I., Sandvik, L., & Ormstad, H.	2021	Norway	cross-sectional study	116	>65 years	older adults applying for public home care service for the first time	1) early detection of frailty;<br>2) estimation of the prevalence of frailty	1) Fried Frailty Phenotype (FFP)<br>2) Short Physical Performance Battery (SPPB)<br>3) Centre for Epidemiological Studies-Depression (CES-D) Scale<br>4) short version International Physical Activity Questionnaire (IPAQ)<br>5) Gait Speed<br>6) Handgrip Strength<br>7) Chair Stand Test (CST)	1) psychosocial well-being<br>2) functional status<br>3) nutritional status<br>4) health behaviour	**1) psychosocial well-being:** depressive symptoms, exhaustion;<br>**2) functional status:** physical function _ gait speed, handgrip strength, leg endurance<br>**3) nutritional status:** weight loss;<br>**4) health behaviour:** level of physical activity	1) the prevalence of frailty among older adults who are applying for public home care services for the first time is significant;<br><br>2) screening for frailty should be considered before older adults apply for public home care service for the first time;<br><br>3) a gait speed threshold of 0.8 m/s could serve as a suitable screening method for identifying frailty in individuals over 70 years old within the general population

No	Study	Authors	Publication year	Country	Study design	Sample size	Age group	Sample characteristics	Study focus	Assessment instruments	Assessment domains	Measurements	Main findings
14	Safety risks among frail older people living at home in the Netherlands - A cross-sectional study in a routine primary care sample	Lette, M., Stoop, A., Nijpels, G., Baan, C., de Bruin, S., & van Hout, H.	2022	Netherlands	cross-sectional study	824	>65 years	frail older adults living at home	examination of the prevalence of risks in multiple domains of life	1) Resident Assessment Instrument for Home Care (interRAI-HC)	1) health conditions<br>2) psychosocial well-being<br>3) cognition<br>4) functional status<br>5) nutritional status<br>6) social support<br>7) environmental assessment<br>8) health behaviours	**1) health conditions:** clinical status-cardio-respiratory health, falls, pain, faecal incontinence, urinary incontinence, pressure ulcer;<br>**2) psychosocial well-being:** mood;<br>**3) cognition:** cognitive functioning, delirium;<br>**4) functional status:** ADLs and IADLs;<br>**5) nutritional status:** dehydration;<br>**6) social support:** abusive relationship, informal care, social functioning;<br>**7) environmental assessment:** home environment;<br>**8) health behaviours:** physical activity, smoking and drinking	the most prevalent risks were associated with individuals' clinical conditions (such as cardio-respiratory health, urinary incontinence, and pain), their functional abilities (including limitations in IADLs and mood), and their social environment (like restrictions in informal care and social functioning)

No	Study	Authors	Publication year	Country	Study design	Sample size	Age group	Sample characteristics	Study focus	Assessment instruments	Assessment domains	Measurements	Main findings
15	A more comprehensive investigation of disability and associated factors among older adults receiving home-based care in rural Dongguan, China	Liang, Y. P., Xu, X. J., Yin, M. J., Li, Y. L., Zhang, Y., Huang, L. F., & Ni, J. D.	2018	China	cross-sectional study	819	≥ 60 years	older adults registered within the Dongguan home-based care system	evaluation of disability in older audlts according to the International Classification of Functioning (ICF), Disability and Health Framework guidelines	1) Barthel ADL Index (B-ADL)<br>2) Mini-Cog (MC)<br>3) Cognitive Performance Scale (CPS)<br>4) Intellectual Disability Rating Scale (IDRS)	1) health conditions<br>2) psychosocial well-being<br>3) cognition<br>4) functional status<br>5) social support<br>6) health behaviour	**1) health conditions:** vision and hearing; multimorbidity;<br>**2) psychosocial well-being:** mental status _ aggressive behaviour and depressive symptoms;<br>**3) cognition:** orienting time and space;<br>**4) functional status:** ADLs (10 activities: eating, bathing, dressing, grooming, controlling bowel function, controlling bladder function, using a toilet unaided, transferring, walking and stair climbing) and IADLs (communication, independence in living, performing, work)<br>**5) social support:** social involvement, social interactions and interpersonal relationships;<br>**6) health behaviour:** the physical activity in hours per week, smoking status and alcohol consumption, the number of hours viewing TV per day	a) impairment of ADL, sensory perception, mental status or social involvement increased the likelihood of risk of the co-occurrence of other deficits;<br><br>b) comprehensive disability in older adults receiving home-based care is linked to factors such as age, inactivity, levels of physical activity, and television viewing

No	Study	Authors	Publication year	Country	Study design	Sample size	Age group	Sample characteristics	Study focus	Assessment instruments	Assessment domains	Measurements	Main findings
16	Protocol of a case-control longitudinal study (fraXity) assessing frailty and complexity among Swiss home service recipients using interRAI-HC assessments	Ludwig, C., & Busnel, C.	2019	Switzerland	prospective observational case-control longitudinal study	70	>65 years	recipients of formal home care (case 1), of formal home assistance (case 2) and individuals free of formal home services (controls)	1) comprehensive geriatric assessments; 2) early screening of frailty and complexity appears to be most relevant	1) Resident Assessment Instrument for Home Care (interRAI-HC) _ Canadian French version 9.1 2) Life History Calendar (LHC) 3) EuroQoL EQ-5D-3L 4) Mini Nutritional Assessment–Short Form (MNA-SF) 5) Montreal Cognitive Assessment (MoCA) 6) Context-based Multiinvariant Detection (COMID) 6) International Consortium for Health Outcomes Measurement (ICHOM)	1) health conditions 2) psychosocial well-being 3) cognition 4) functional status 5) nutritional status 6) social support 7) environmental assessment 8) health behaviour 9) responsibility 10) quality of life	**1) health conditions:** sensory abilities (vision and hearing), continence, medical diagnoses, falls, physical abilities, physical symptoms and pain, skin and feet problems, medications, ongoing therapies; **2) psychosocial well-being:** mood (self-perceived isolation and loneliness); **3) cognition:** cognitive functioning **4) functional status:** ADLs; **5) nutritional status** **6) social support:** formal and informal care, advanced care instructions, social participation and caregiving; **7) environmental assessment:** living conditions, home environment; **8) health behaviour:** health-related behaviour, social behaviour; **9) responsibility:** legal representativity; **10) quality of life:** self-perceived health-related quality of life, overall satisfaction with life	1) the expected outcomes of the fraXity study include a) reliable algorithms for calculating frailty and complexity scores based on the interRAI-HC, and b) clinical assessment protocols for homecare nurses; 2) these results should provide essential stakeholders in the healthcare system with the tools to enhance their roles in a joint effort to deliver optimal care and improve the quality of life for older adults

No	Study	Authors	Publication year	Country	Study design	Sample size	Age group	Sample characteristics	Study focus	Assessment instruments	Assessment domains	Measurements	Main findings
17	Use of the resident assessment instrument to measure depression in indigenous home care clients	Maranzan, K. A., & Stones, M. J.	2013	Canada	cross-sectional study	126423	all age group	indigenous people	1) examination of sociodemographic influences on depression and assessed for home care or potential long-term care admission; 2) assessment of eligibility for home care	1) Resident Assessment Instrument for Home Care (interRAI-HC) 2) Cognitive Performance Scale (CPS) 3) Depression Rating Scale (DRS) 4) Changes in Health, End-Stage Disease and Symptoms and Signs (CHESS) Scale	1) health conditions 2) psychosocial well-being 3) cognition 4) functional status	**1) health conditions** **2) psychosocial well-being:** mood, depressive symptoms; **3) cognition:** cognitive functioning _ decline in cognition; **4) functional status:** instability in health and frailty, deterioration in ADLs	clients who were female, younger in age, and had lower levels of education exhibited higher depression scores, as did those with poorer health conditions
18	Assessment of nutritional status of elderly receiving home health care	Meriç, C. S., Ayhan, N. Y., & Yilmaz, H. O.	2022	Turkey	cross-sectional study	120	>65 years	older adults who were registered to Home Health Service	evaluation of the nutritional status of elderly who received home health care	Mini Nutritional Assessment-Short Form (MNA-SF)	nutritional status	nutritional status	the nutritional status of elderly individuals receiving home health care should be assessed regularly by dietitians

No	Study	Authors	Publication year	Country	Study design	Sample size	Age group	Sample characteristics	Study focus	Assessment instruments	Assessment domains	Measurements	Main findings
19	Nursing care needs and services utilised by home-dwelling elderly with complex health problems: observational study	Naess, G., Kirkevold, M., Hammer, W., Straand, J., & Wyller, T. B.	2017	Norway	observational study	-	>75 years	– age 75 years or more<br>– home-dwelling<br>– receiving home care on a daily basis<br>– three or more chronic diagnoses recorded in the home nursing service's patient record, and<br>– hospitalized at least once during the last year	Identification of needs older adults for nursing interventions and how these needs were met	1)Trail Making (TM) Test<br>2) Timed Up and Go (TUG) Test<br>3) Barthel ADL Index (B-ADL)	1) cognition<br>2) functional status	**1) cognition:** cognitive functioning;<br>**2) functional status:** mobility, handgrip strength, independence in primary ADL tasks	for the extremely frail population, home care resources should likely be utilized more flexibly and proactively to focus on maintaining functional ability, reducing symptom burden, and preventing unnecessary hospitalizations

No	Study	Authors	Publication year	Country	Study design	Sample size	Age group	Sample characteristics	Study focus	Assessment instruments	Assessment domains	Measurements	Main findings
20	Evaluation of malnutrition frequency and related factors of geriatric patients in need of home healthcare	Ozkoc, M. N. S., & Ardic, C.	2023	Turkey	cross-sectional descriptive study	161	>65 years	older adults who were registered in the Home Healthcare Unit	determination of nutritional status of frail older adults receiving home health services	1) Mini Nutritional Assessment (MNA)<br>2) Mini Nutritional Assessment Short Form (MNA-SF)	nutritional status	**nutritional status:**<br>1) 6 questions on food intake, weight loss status, mobility, acute illness/stress, neuropsychological status, and body mass index (BMI);<br>2) 12 questions concerning dependency, medication usage, pressure sores, food and drink intake, number of meals, nutritional autonomy, self-perception of health and nutrition, and arm and calf circumference	individuals who were fed by caregivers tended to be more malnourished compared to those who were able to feed themselves

No	Study	Authors	Publication year	Country	Study design	Sample size	Age group	Sample characteristics	Study focus	Assessment instruments	Assessment domains	Measurements	Main findings
21	Assessment without action; A randomised evaluation of the inter RAI home care compared to a national assessment tool on identification of needs and service provision for older people in New Zealand	Parsons, M., Senior, H., Chen, X. M. H., Jacobs, S., Parsons, J., Sheridan, N., & Kenealy, T.	2013	New Zealand	randomised controlled trial	316	>65 years	community-dwelling older adults	comparison of the interRAI-HC with the SNA in community-dwelling older adults	1) Resident Assessment Instrument for Home Care (interRAI-HC)<br>2) Support Needs Assessment (SNA)<br>3) SF-36<br>4) Barthel ADL Index (B-ADL)<br>5) Nottingham Extended ADL Scale<br>6) Dukes Social Support Index (DSSI)<br>7) Abbreviated Mental Test Score (AMTS)<br>8) Geriatric Depression Scale (GDS-15)	1) health conditions<br>2) psychosocial well-being<br>3) cognition<br>4) functional status<br>5) social support<br>6) financial assessment	**1) health conditions:** general health status;<br>**2) psychosocial well-being:** mood, depressive symptoms;<br>**3) cognition:** cognitive status;<br>**4) functional status:** functional abilities _ housekeeping, personal care, mobility, ADLs and IADLs;<br>**5) social support:** informal support, social support and support service use;<br>**6) financial assessment:** income	1) outcomes included health-related quality of life, physical function, social support, cognitive status, mood and health service usage as well as identified need;<br><br>b) more social and carer support were recommended by the SNA and more rehabilitation and preventive health screens were recommended by the interRAI-HC

No	Study	Authors	Publication year	Country	Study design	Sample size	Age group	Sample characteristics	Study focus	Assessment instruments	Assessment domains	Measurements	Main findings
22	Assessing physical performance and physical activity in large population-based aging studies: Home-based assessments or visits to the research center?	Portegijs, E., Karavirta, L., Saajanaho, M., Rantalainen, T., & Rantanen, T.	2019	Finland	cross-sectional study	1886	older adults aged 75, 80, and 85 years	Older adults in a home versus a highly standardized setting of the research centre	comparison of correlations between a range of measures of physical performance and physical activity assessing the same underlying construct in different settings, that is, in a home versus a highly standardized setting of the research centre or accelerometer recording	1) Yale Physical Activity Survey<br>2) Mini Mental State Examination (MMSE)<br>3) Centre for Epidemiologic Studies for Depression (CES-D) Scale<br>4) Assessment of ADLs, instrument not specified<br>5) Gait Speed<br>6) Handgrip Strength	1) health conditions<br>2) psychosocial well-being<br>3) cognition<br>4) functional status<br>5) nutritional status<br>6) social support<br>7) environmental assessment<br>8) health behaviour<br>9) financial assessment	**1) health conditions**<br>**2) psychosocial well-being:** depressive symptoms;<br>**3) cognition:** cognitive functions;<br>**4) functional status:** walking speed, handgrip strength; ADLs (feeding, rising from or lying down on a bed, dressing, bathing, and toileting);<br>**5) nutritional status**<br>**6) social support:** neibourhood interaction;<br>**7) environmental assessment:** living situation;<br>**8) health behaviour:** self-reported physical activity;<br>**9) financial assessment:** financial situation	it is possible to include older adults experiencing early declines in health, function, and overall activity in studies focused on physical performance and physical activity

No	Study	Authors	Publication year	Country	Study design	Sample size	Age group	Sample characteristics	Study focus	Assessment instruments	Assessment domains	Measurements	Main findings
23	Global functionality and associated factors in the older adults followed by Home Care in Primary Health Care	Predebon, M. L., Ramos, G., Pizzol, F. L. F. D., Santos, N. O. D., Paskulin, L. M. G., & Rosset, I.	2021	Brazil	cross-sectional study	124	≥60 years	older adults followed by the HC1 of the CSD in the city of Porto Alegre	analysis of the association of global functionality with the main functional systems and the sociodemographic variables	1) Barthel ADL Index (B-ADL)<br>2) Lawton IADL Scale (L-IADL)<br>3) Mini Mental State Exam (MMSE0<br>4) Timed Up and Go (TUG) Test<br>5) Geriatric Depression Scale (GDS-15)	1) psychosocial well-being<br>2) cognition<br>3) functional status<br>4) social support<br>5) financial assessment	**1) psychosocial well-being:** mood, depressive symptoms;<br>**2) cognition:** communication;<br>**3) functional status:** functionality-ADLs and IADLs, mobility;<br>**4) social support:** living arrangements (living alone or with a companion);<br>**5) financial assessment:** family income	the decline in cognitive and mobility was associated with poor functionality in basic and instrumental activities of daily living
24	Sociodemographic factors affecting older people's care dependency in their daily living environment according to Care Dependency Scale (CDS)	Puto, G., Sowinska, I., Scislo, L., Walewska, E., Kaminska, A., & Muszalik, M.	2021	Poland	cross-sectional study	151	>65 years	Old adults followed by the HC1 of the CSD in the city of Porto	Determination of the influence of sociodemographic factors on the care dependency of older adults	1) Abbreviated Mental Test Score (AMTS)<br>2) Care Dependency Scale (CDS)<br>3) Katz Index of Independence in ADLs (K-ADL)<br>4) Lawton IADL Scale (L-IADL)<br>5) Mini Nutritional Assessment (MNA)<br>6) Geriatric Depression Scale (GDS)	1) health conditions<br>2) psychosocial well-being<br>3) functional status<br>4) nutritional status<br>5) social support<br>6) responsibility	**1) health conditions**<br>**2) psychosocial well-being:** subjective sense of being depressed;<br>**3) functional status:** ADLs (bathing, dressing, going to toilet, transferring from bed to a chair, feeding, and continence) and IADLs (ability to use the telephone, mode of transportation, food preparation, housekeeping (cleaning, house maintenance, and laundering), responsibility for one's own medication, and ability to handle finances), mobility;<br>**4) nutritional status:** assessment of the risk of malnutrition;<br>**5) social support:** social contact (involvement);	sociodemographic factors greatly influence the degree of care dependency. For example, gender (22.4% for females vs. 6.1% for males), age (older individuals), marital status (being married), and a higher level of education are all associated with a medium to high level of dependency

No	Study	Authors	Publication year	Country	Study design	Sample size	Age group	Sample characteristics	Study focus	Assessment instruments	Assessment domains	Measurements	Main findings
												**6) responsibility:** sense of rules and values	

25	Comprehensive clinical assessment of home-based older persons within New Zealand: An epidemiological profile of a national cross-section	Schluter, P. J., Ahuriri-Driscoll, A., Anderson, T. J., Beere, P., Brown, J., Dalrymple-Alford, J., David, T., Davidson, A., Gillon, D. A., Hirdes, J., Keeling, S., Kingham, S., Lacey, C., Menclova, A. K., Millar, N., Mor, V., & Jamieson, H. A.	2016	New Zealand	cross-sectional study	51532	all age group; >65 years (96.2%)	Alegre.	assessment of its data quality and evaluation of its ability to be matched	1) Home Care International Residential Assessment Instrument (interRAI-HC)<br>2) Hamilton Depression Rating Scale (HDRS)<br>3) Cornell Scale for Depression in Dementia (CSDD)<br>4) Depression Rating Scale (DRS)<br>5) Changes in Health, End-stage disease and Signs and Symptoms (CHESS) Scale<br>6) Barthel ADL Index (B-ADL)	**20 Domains:**<br>A: identification information<br>B: intake and initial history<br>C: cognition<br>D: communication and vision<br>E: mood and behaviour<br>F: psychosocial well-being<br>G: functional status<br>H: continence<br>I: disease diagnoses<br>J: health conditions<br>K: oral and nutritional status<br>L: skin condition<br>M: medications<br>N: treatment and procedures<br>O: responsibility<br>P: social support<br>Q: environmental assessment<br>R: discharge potential and overall status<br>S: discharge<br>T: assessment Information	A: identification information<br>B: intake and initial history<br>C: cognition<br>D: communication and vision<br>E: mood and behaviour<br>F: psychosocial well-being<br>G: functional status<br>H: continence<br>I: disease diagnoses<br>J: health conditions<br>K: oral and nutritional status<br>L: skin condition<br>M: medications<br>N: treatment and procedures<br>O: responsibility<br>P: social support<br>Q: environmental assessment<br>R: discharge potential and overall status<br>S: discharge<br>T: assessment Information	this national database offers researchers a valuable opportunity to gain deeper insights into the health and healthcare of older adults, helping to better support them in remaining in their own homes

No	Study	Authors	Publication year	Country	Study design	Sample size	Age group	Sample characteristics	Study focus	Assessment instruments	Assessment domains	Measurements	Main findings
26	Measures of frailty in homebound older adults	Shirai, S., Kwak, M. J., & Lee, J.	2022	US	retrospective case review	25	53-90 years	homebound older adults	evaluation of the prevalence of frailty	1) Fried Frailty Phenotype (FFP)	1) functional status<br>2) nutritional status<br>3) health behaviours	**1) functional status:** weakness (handgrip strength), poor endurance, slowness (gait speed);<br>**2) nutritional status:** unintentional weight loss;<br>**3) health behaviours:** physical activity levels	frailty is common among older adults who are homebound and may be associated with reduced walking speed, polypharmacy, and/or multimorbidity
27	Assessing sarcopenia, frailty, and malnutrition in community-dwelling dependant older adults- An exploratory home-based study of an underserved group in research	Swan, L., Martin, N., Horgan, N. F., Warters, A., & O'Sullivan, M.	2022	Ireland	exploratory study	31	>65 years	community-dwelling older adults in receipt of state-funded home care	exploration of the assessment of sarcopenia, frailty, and malnutrition	1) Handgrip Strength<br>2) Chair Rise Test (CRT)<br>3) SARC-F tool<br>4) Mini Nutritional Assessment (MNA)<br>5) Clinical Frailty Scale (CFS)<br>6) International Physical Activity Questionnaire (IPAQ)<br>7) Barthel ADL Index (B-ADL)	1) health conditions<br>2) cognition<br>3) functional status<br>4) nutritional status<br>5) health behaviours	**1) health conditions:** functional comorbidity, number of medications, falls;<br>**2) cognition**<br>**3) functional status:** ADLs; sarcopenia, weakness (handgrip strength), poor endurance, slowness (gait speed), assistance in walking;<br>**4) nutritional status:** unintentional weight loss;<br>**5) health behaviours:** physical activity levels, smoking and alcohol consumption	most older adults living in the community who receive home support were at risk of developing probable sarcopenia, frailty, and low levels of physical activity, with more than 25% also at risk of malnutrition

No	Study	Authors	Publication year	Country	Study design	Sample size	Age group	Sample characteristics	Study focus	Assessment instruments	Assessment domains	Measurements	Main findings
28	Health status and individual care needs of disabled elderly at home in different types of care	Tang, Q., Yuan, M., Wu, W., Wu, H., Wang, C., Chen, G., Li, C., & Lu, J.	2022	China	cross-sectional study	559	>60 years	disabled older adults	exploration of the basic characteristics and health status, discovering the survival problems of older adults, while meeting their care needs	1) Kalz ADL Index<br>2) Lawton ADL Index<br>3) General Health Questionnaire (GHQ)<br>4) 12-item Short Form Survey (SF–12)<br>5) Social Network Analysis (SNA)	1) health conditions<br>2) psychosocial well-being<br>3) functional status<br>4) social support<br>5) health behaviours<br>6) quality of life<br>7) care needs	**1) health conditions:** health status _ chronic diseases, the number of diagnoses, and main diseases;<br>**2) psychosocial well-being:** mental health of the disabled elderly<br>**3) functional status:** ADLs and IADLs<br>**4) social support:** social networks<br>**5) health behaviours:** smoking, drinking, and taking exercise;<br>**6) quality of life;**<br>**7) individual care needs**: basic living support (BLS), medical and nursing support (MNS), social participation support (SPS), and psychological support(PS)]	a) the care needs of the disabled elderly are diversified, of which a vast majority of them have not been fully guaranteed;<br><br>b) there is an urgent need to enhance the accuracy of identifying care needs for elderly individuals with disabilities, along with the development of detailed and personalized care plans for them

No	Study	Authors	Publication year	Country	Study design	Sample size	Age group	Sample characteristics	Study focus	Assessment instruments	Assessment domains	Measurements	Main findings
29	Chair rise capacity and associated factors in older home-care clients	Tiihonen, M., Hartikainen, S., & Nykänen, I.	2018	Finland	population-based multidisciplinary intervention study	267	>75 years	home-care clients	investigation of the ability of older home-care clients to perform the five times chair rise test and associated personal characteristics, nutritional status and functioning	1) Barthel ADL Index (B-ADL)<br>2) Lawton IADL Scale (L-IADL)<br>3) Mini Mental State Examination (MMSE)<br>4) Geriatric Depression Scale (GDS-15)<br>5) Mini Nutritional Assessment (MNA)<br>6) Functional Comorbidity Index (FCI)<br>7) Chair Stand Test (CST)	1) health conditions<br>2) psychosocial well-being<br>3) cognition<br>4) functional status<br>5) nutritional status<br>6) social support	**1) health conditions:** physical comorbidity, medical diagnoses and medications;<br>**2) psychosocial well-being:** mood, depressive symptoms;<br>**3) cognition:** cognitive functioning;<br>**4) functional status:** functional abilities _ ADLs (basic ADLs, such as eating, washing, getting around and sphincter control) and IADLs (using the telephone, grocery shopping, preparation of meals, housekeeping, doing laundry, mode of transportation, taking care of medications and managing money);<br>**5) nutritional status**<br>**6) social support:** living arrangements (living alone or with companion)	1) impaired functional mobility, often linked to lower education levels, multiple comorbidities, and inadequate nutritional status, was frequently observed among elderly home care clients;<br><br>2) physical training and nutritional services are necessary to preserve functional mobility and prevent further deterioration
30	Participation needs of older adults having disabilities and receiving home care: Met needs mainly concern daily activities, while unmet needs mostly involve social activities	Turcotte, P.-L., Lariviere, N., Desrosiers, J., Voyer, P., Champoux, N., Carbonneau, H., Carrier, A., & Levasseur, M.	2015	Canada	qualitative study _ multiple case study design	33	66-88	older adults receiving home care services from a HSSC	exploration of the participation needs	Functional Autonomy Measurement System (FAMS)	1) health conditions<br>2) psychosocial well-being<br>3) cognition<br>4) functional status<br>5) nutritional status<br>6) social support<br>7) financial assessment	**1) health conditions:** type of disability and health problems, and time since onset of health problems;<br>**2) psychosocial well-being:** mental functions;<br>**3) cognition:** communication<br>**4) functional status:** mobility, ADLs and IADLs;<br>**5) nutritional status**<br>**6) social support:** interpersonal relationships, residential status, living arrangements;<br>**7) financial assessment:** income	effectively evaluating and meeting these needs is essential for enhancing the health and well-being of older adults

No	Study	Authors	Publication year	Country	Study design	Sample size	Age group	Sample characteristics	Study focus	Assessment instruments	Assessment domains	Measurements	Main findings
31	Major depression and subthreshold depression among older adults receiving home care	Xiang, X. L., Leggett, A., Himle, J. A., & Kales, H. C.	2018	US	cross-sectional design	811	>60 years	community-dwelling older adults who received paid home care	estimation of the prevalence and correlates of major and subthreshold depression and the extent of treatment utilization in older adults receiving home care	1) Composite International Diagnostic Interview Short Form (CIDI-SF)<br>2) Centre for Epidemiologic Studies Depression Scale (CES-D)	psychosocial well-being	**psychosocial well-being:** depressive symptoms, sleeping functions, mood, satisfaction with life, sense of loneliness, feelings of sadness, experiences of dysphoria and/or anhedonia	1) depression impacts a significant number of older adults who are receiving home care and may not be treated appropriately;<br><br>2) further research is necessary to create effective strategies for incorporating depression assessment and treatment into home care

No	Study	Authors	Publication year	Country	Study design	Sample size	Age group	Sample characteristics	Study focus	Assessment instruments	Assessment domains	Measurements	Main findings
32	The level of independence in daily functioning of the elderly in home environments	Zboina, B., Slusarska, B., Stepien, R., Nowicki, G., & Wiraszka, G.	2018	Poland	cross-sectional design	401	>65 years	Older adults with full logical and verbal contact with the subject	determination of the functional capacity and independence in daily functioning	EASY-Care questionnaire (1999-2002 Polish version);	1) health conditions<br>2) psychosocial well-being<br>3) cognition<br>4) functional status<br>5) nutritional status<br>6) social support<br>7) environmental assessment	**1) health conditions:** vision and hearing;<br>**2) psychosocial well-being:** emotional status (sense of loneliness);<br>**3) cognition:** communication;<br>**4) functional status:** self-care, walking outside, ADLs and IADLs;<br>**5) nutritional status:** ability to chew;<br>**6) social support:** identification of individuals providing help with individual activities;<br>**7) environmental assessment:** housing conditions	1) the most commonly reported issues were related to eyesight and hearing problems;<br><br>2) subjects with cardiovascular and respiratory disorders exhibited notably reduced levels of functional capacity

Supplementary Table S3. Average MMAT results for studies evaluated in the review.

No	Study	Quality level		
		High	Moderate	Low
1	Abreu et al. (2019)	5		
2	Ballard et al. (2013)	5		
3	Bardstu et al. (2022)	5		
4	Berggren et al. (2020)		3.5	
5	Black et al. (2013)	5		
6	Dale et al. (2018)	4.5		
7	Davidson & Guthrie (2019)	5		
8	Engelheart et al. (2021)		3.5	
9	Garms-Homolova et al. (2017)	5		
10	Grimsland et al. (2019)		4	
11	Helvik et al. (2019)	5		
12	Kiesswetter et al. (2013)		3	
13	Laukli et al. (2021)	4.5		
14	Lette et al. (2022)	5		
15	Liang et al. (2018)	5		
16	Ludwig & Busnel (2019)	5		
17	Maranzan & Stones (2013)		4	
18	Meriç et al. (2022)		2.5	
19	Naess et al. (2017)	5		
20	Ozkoc & Ardic (2023)		3	
21	Parsons et al. (2013)	5		
22	Portegijs et al. (2019)	5		
23	Predebon et al. (2021)		3.5	
24	Puto et al. (2021)	4.5		
25	Schluter et al. (2016)	5		
26	Shirai et al. (2022)		3.5	
27	Swan et al. (2022)	5		
28	Tang et al. (2022)	5		
29	Tiihonen et al. (2018)	5		

30	Turcotte et al. (2015)	5	
31	Xiang et al. (2018)	5	
32	Zboina et al. (2018)		2
